# Comparative Analysis of DNA Nanoparticles and AAVs for Ocular Gene Delivery

**DOI:** 10.1371/journal.pone.0052189

**Published:** 2012-12-18

**Authors:** Zongchao Han, Shannon M. Conley, Rasha Makkia, Junjing Guo, Mark J. Cooper, Muna I. Naash

**Affiliations:** 1 Department of Cell Biology, University of Oklahoma Health Sciences Center, Oklahoma City, Oklahoma, United States of America; 2 Copernicus Therapeutics, Inc., Cleveland, Ohio, United States of America; Cedars-Sinai Medical Center, United States of America

## Abstract

Gene therapy is a critical tool for the treatment of monogenic retinal diseases. However, the limited vector capacity of the current benchmark delivery strategy, adeno-associated virus (AAV), makes development of larger capacity alternatives, such as compacted DNA nanoparticles (NPs), critical. Here we conduct a side-by-side comparison of self-complementary AAV and CK30PEG NPs using matched ITR plasmids. We report that although AAVs are more efficient per vector genome (vg) than NPs, NPs can drive gene expression on a comparable scale and longevity to AAV. We show that subretinally injected NPs do not leave the eye while some of the AAV-injected animals exhibited vector DNA and GFP expression in the visual pathways of the brain from PI-60 onward. As a result, these NPs have the potential to become a successful alternative for ocular gene therapy, especially for the multitude of genes too large for AAV vectors.

## Introduction

Recombinant adeno-associated viruses (AAVs) have been highly successful for ocular gene therapy due to their safety and ability to drive long-term gene expression [1], [2], [3], [4]. AAV-based clinical trials for RPE65-associated Leber congenital amaurosis [4], [5], [6], [7] have reported no significant side effects and some positive visual outcomes [4], [6], [7]. As a result of these trials and numerous animal studies [3], [8] AAV is considered the current leading vector system for ocular gene therapy. Generally AAVs do not exhibit many of the problems suffered by other viral vectors in the eye, such as induction of severe immune responses and insertional mutagenesis, although they can have adverse interactions with viruses pre-existing in the target tissue [9], [10], [11], [12]. Furthermore, AAVs do not share traditional limitations of non-viral vectors such as transient gene expression and low cellular uptake. However, while AAVs are well-positioned to remain key players for ocular gene therapy, their limited vector capacity prevents them from being useful for the delivery of large genes, such as ABCA4 and USH2A. Although recent studies have experimented with ways to increase AAV capacity [13], [14], the results are controversial and inconclusive underscoring the need for alternative tools for ocular gene therapy.

In contrast to many other non-viral delivery options, DNA nanoparticles (NPs) composed of single molecules of DNA compacted with 10 kDa polyethylene glycol-substituted polylysine (CK30PEG) drive efficient, persistent retinal gene expression [15], [16]. These small NPs (minor diameter of 8–11 nm) are efficiently taken up into dividing and non-dividing cells and remain episomal [17], [18]. They were safe and effective in a human clinical trial for cystic fibrosis and are currently being employed in the lung, brain, and eye [15], [16], [19], [20], [21], [22], [23]. We have demonstrated that NPs can be safely used to target the photoreceptors and RPE cells without significant toxicity [21], [24], [25] and mediate improvement in the retinitis pigmentosa phenotype of the retinal degeneration slow (*rds^+/−^)* mouse model [15], [16]. Critically, these NPs drive efficient gene expression with vectors up to 20 kbp (the largest tested) which make them an ideal complement to AAVs especially for delivery of large genes [26].

To formally assess the ability of NPs to function as a relevant therapeutic option for monogenic ocular diseases, we here conducted side-by-side studies comparing reporter gene (GFP) expression from self-complementary AAV2 (the serotype currently being used in clinical trials [4], [6], [7]) and self-complementary AAV5 (highly efficient for ocular delivery [27], [28]) with that from CK30PEG NPs generated from the same ITR plasmids. We chose to test two different promoters, the ubiquitously expressed chicken β-actin (CBA) promoter and the photoreceptor-specific (rod and cone [15], [29], [30]) mouse opsin promoter (MOP) since both have been used successfully in a variety of gene therapy studies [7], [10], [15], [16], [29], [31], [32]. Here we show that DNA NPs can drive reporter gene expression on the same scale and duration as AAV, emphasizing their therapeutic potential for ocular diseases, especially those associated with defects in large genes.

## Results

### Nanoparticles can Generate Persistent Gene Expression at Levels Comparable to AAV

To compare expression levels from AAVs with those from NPs, we first conducted a dose-response study. Identical plasmids (either pscCBA-GFP or pscMOP-GFP) were used to produce AAVs or acetate-formulated CK30PEG10K NPs (AAV2-CBA-GFP, NP-CBA-GFP, AAV5-MOP-GFP, NP-MOP-GFP), and in some experiments uncompacted (naked) plasmids were used as controls.

**Figure 1:**
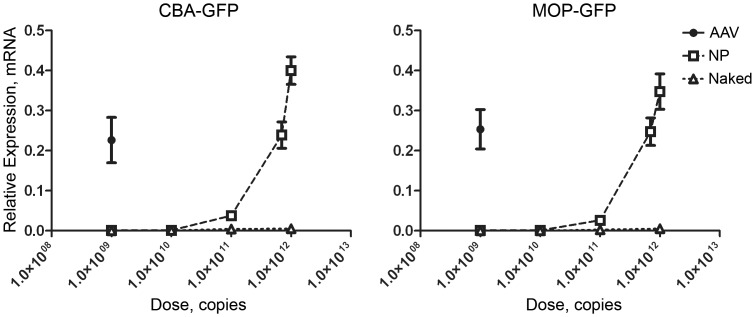
NPs drive gene expression at a comparable scale to AAVs. AAV2-CBA-GFP, AAV5-MOP-GFP, NP-CBA-GFP, NP-MOP-GFP, Naked-CBA-GFP, or Naked-MOP-GFP were subretinally delivered in 1 µl at the indicated dose to P30 Balb/C mice (1.45 µl for NP at 10^12^). Whole eyes were collected at PI-14 and GFP expression was measured by qRT-PCR. Values are normalized to β-actin. N = 6/cohort, shown are means ± SEM.

Mice at post-natal day (P) 30 were bilaterally subretinally injected with 1 µl of either 10^9^ vg of AAVs or 10^9^–10^11^ vg of NPs. For the highest dose of NPs (10^12^), the injection volume was 1.45 µl (due to the concentration of the NPs). Eyes were collected at post-injection day (PI-) 14 and GFP message levels were analyzed by qRT-PCR. NPs at a dose of 6.9^11^ vg yielded GFP expression levels comparable to those from AAVs at 10^9^ vg (**Fig. 1** difference not significant by Student’s t-test). At the 10^12^ dose, NP-CBA-GFP drove significantly higher gene expression than AAV2-CBA-GFP at 10^9^ vg (p = 0.02 by Student’s t-test). NP-MOP-GFP levels at 10^12^ were also higher than AAV5-MOP-GFP at 10^9^, but the difference was not significant. The 6.9^11^ vg dose corresponds to 4.3 µg/µl, a NP concentration we have previously used to drive optimal ocular gene expression [15], [16], [24], [25]. 10^9^ vg of AAV was chosen as the reference amount as this quantity of AAV has been safely used in many ocular studies [33], [34], [35]. Consistent with the previously described lack of toxicity with these doses, we saw no macrophage/neutrophil infiltration (**Fig. S1**) or elevation of inflammatory cytokines (not shown) with any group at PI-14. No GFP was detected from naked DNA or saline treated eyes at PI-14. For the remainder of this study, 4.3 µg of NPs/naked DNAs and 10^9^ vg of AAVs were delivered in 1 µl.

**Figure 2:**
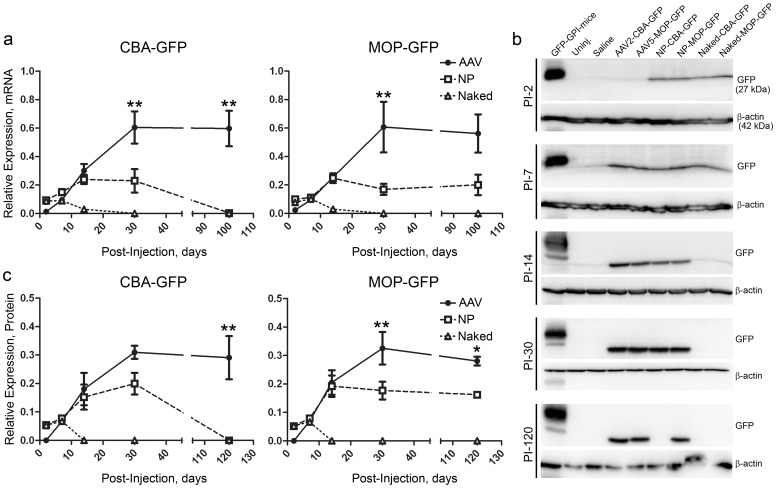
NP- and AAV-mediated gene expression persisted for up to four months. P30 Balb/C mice were subretinally injected with Naked-CBA-GFP, Naked-MOP-GFP, NP-CBA-GFP, NP-MOP-GFP (6.9^11^ vg), AAV2-CBA-GFP, or AAV5-MOP-GFP (10^9^ vg). **a.** GFP mRNA levels (normalized to β-actin ) in whole eyes were assessed by qRT-PCR at the indicated timepoints. **B.** Shown are representative western blots probed with antibodies against GFP/actin to assess GFP protein expression in the retina at various timepoints PI. **c.** Densitometric quantification of western blots represented in (**b**). GFP band densities were quantitated, normalized to β-actin, and expressed as a fraction of that found in GPI-GFP transgenic mice (used as a positive control in every blot). **a, c.** N = 6/cohort, shown are means ± SEM *p<0.01, **p<0.001 for comparsion between AAV and NP, by two-way ANOVA with Bonferroni’s post-hoc test.

To assess the onset/persistence of gene expression from AAVs and NPs, GFP expression was measured in whole eyes by qRT-PCR (**Fig. 2a**) and in retinas by western blot (**Fig. 2b–c**) at multiple timepoints. GFP in naked DNA and NP treated eyes was detected at PI-2 (earliest timepoint evaluated) while expression in eyes injected with AAVs was not seen until PI-7. No gene expression was detected in naked DNA injected eyes after PI-7. NP and AAV-injected eyes exhibited similar message and protein levels at PI-7 and PI-14 after which levels in NP-injected eyes leveled off while levels in AAV-treated eyes continued to rise. At PI-30, message levels were ∼3-fold higher in AAV-treated eyes than NP-treated eyes (**Fig. 2a** **,** **p<0.001 in comparisons between AAV and NP). After PI-30, transgene expression remained stable from all groups except NP-CBA-GFP which exhibited no detectable GFP message or protein. At PI-120, GFP protein levels in retinas from NP-MOP-GFP treated eyes were 58% of levels in AAV-MOP-GFP treated eyes (**Fig. 2c** *p<0.01). mRNA levels at PI-30 and PI-120 in NP-treated eyes were 28–38% of those in AAV-treated eyes while protein levels in NP-treated retinas were 54–64% of those in AAV-treated retinas (depending on promoter and timepoint); however, direct comparisons between protein and mRNA levels should not be made since mRNA levels were from whole eyes while protein levels were from retinas. Although they are less efficient per vg, these data indicate that in common with AAVs, NPs can successfully drive persistent gene expression.

### Distribution of Gene Expression after AAV and NP Treatment

**Figure 3:**
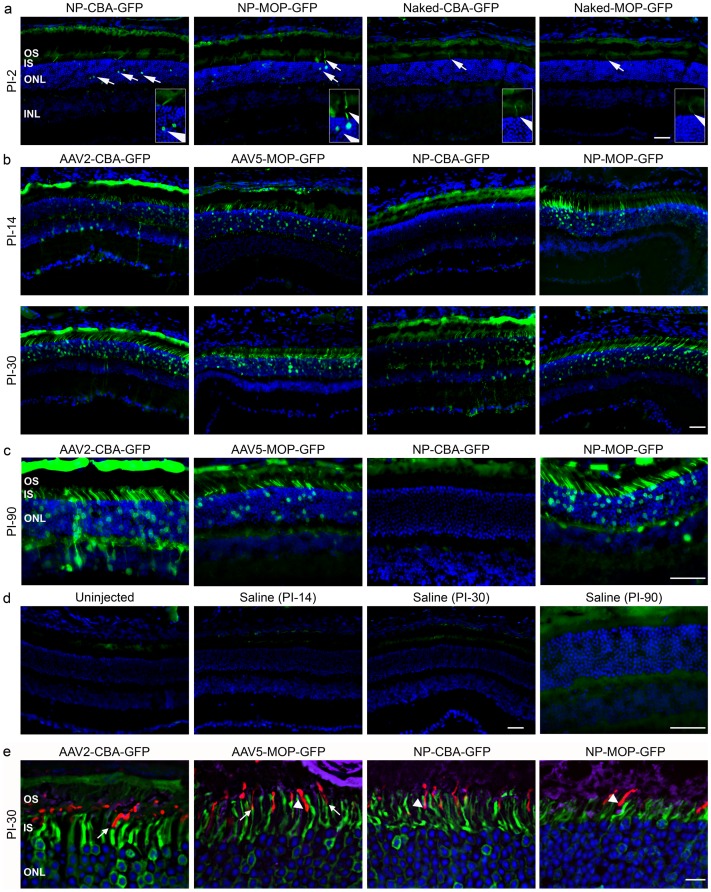
Subretinal delivery of AAVs and NPs efficiently transduces retinal tissues. Balb/C mice were subretinally injected at P30 with Naked-CBA-GFP, Naked-MOP-GFP, NP-CBA-GFP, NP-MOP-GFP (6.9^11^ vg), AAV2-CBA-GFP, or AAV5-MOP-GFP (10^9^ vg). Native GFP fluorescence was imaged using a spinning disk confocal microscope at PI-2 (**a**), PI-14, PI-30 (**b**), and PI-90 (**c**). Negative control images are shown in (**d**). Scale bars: 40 µm. **e**. Sections were labeled with antibodies against rod opsin (purple) and S-opsin (red), and nuclei were counterlabeled with DAPI. Green is GFP native fluorescence. Arrows show cone photoreceptors which express GFP, while arrowheads highlight cones which do not express GFP. N = 3 eyes/group. Scale bar: 10 µm. Shown are representative single planes from confocal stacks. To control for normal retinal autofluorescence, images were captured at equivalent exposure times from control and experimental eyes. OS: outer segment, IS: inner segment, ONL: outer nuclear layer, INL: inner nuclear layer. N = 3–5 eyes/cohort.

**Figure 4:**
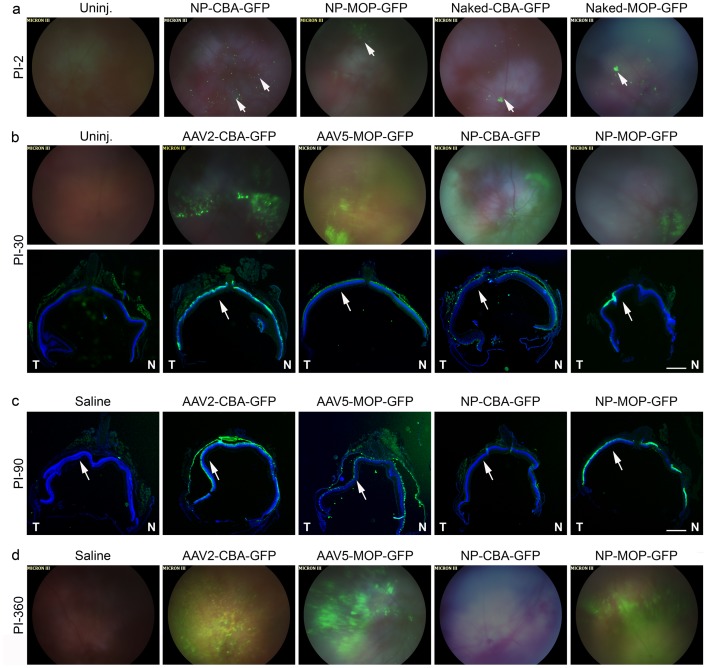
GFP transduced cells are distributed throughout the retina in AAV and NP-treated animals and persist for up to one year. Balb/C mice were subretinally injected at P30 with Naked-CBA-GFP, Naked-MOP-GFP, NP-CBA-GFP, NP-MOP-GFP (6.9^11^ vg), AAV2-CBA-GFP, or AAV5-MOP-GFP (10^9^ vg). GFP distribution was examined *in vivo* by brightfield/GFP fundus imaging at PI-2 (a-arrows show regions of early expression), PI-30 (b-top row), and PI-360 (d). Distribution was also assessed by capturing images of native GFP fluorescence in entire sections cut through the optic nerve at PI-30 (b-bottom row), and PI-90 (c). Arrows in b, c show the approximate region of injection. Scale bar, 500 µm. N-nasal, T-temporal. N = 3–5 eyes/cohort.

To assess distribution of expression after NP and AAV delivery, native GFP fluorescence was examined in retinal sections (**Fig. 3**) and fundus images (**Fig. 4**) at various time-points. Photomicrographs were collected from the temporal central region in order to coincide approximately with the region of injection. At PI-2, no GFP-expressing cells were detected in AAV-injected eyes (not shown), although they were seen in the outer nuclear layer (ONL) of NP-injected eyes (**Fig. 3a** and insets). By PI-14, a significant number of GFP positive cells were apparent from each AAV and NP group. Consistent with the cell-specificity of the promoter, expression from AAV5-MOP-GFP and NP-MOP-GFP was restricted to photoreceptors while AAV2-CBA-GFP and NP-CBA-GFP expressed in the ONL, inner nuclear layer (INL), ganglion cell layer, and retinal pigment epithelium (RPE, **Fig. 3b–c**). No GFP was detected in the cornea, lens, choroid, or sclera from any group, confirming that there was minimal vector leakage into the vitreous during the injection. For MOP-containing vectors, no differences in GFP expression pattern between AAV and NPs were noted at PI-14, PI-30, or PI-90. Consistent with mRNA and protein data from **Fig. 2**, GFP was not detectable in NP-CBA-GFP treated eyes at PI-90 (**Fig. 3c**). Co-labeling with antibodies against S-opsin (red) and rod-opsin (purple, **Fig. 3e**) confirmed GFP was expressed in rods and cones (arrows) although not all cones expressed GFP (**Fig. 3e**, arrowheads).

We then evaluated the distribution of GFP-positive cells in retinal cross-sections and by *in vivo* fundus imaging. **Fig. 4** shows brightfield/GFP fundus images combined, and **Fig. S2** shows fundus images presenting the green channel only. No GFP signal was detected in AAV-treated eyes by fundus imaging at PI-2 (not shown) while low levels of GFP were seen in NP and naked DNA injected eyes (arrows, **Fig. 4a**). At PI-30, fundus imaging (**Fig. 4b**, top) showed GFP throughout the retina in NP and AAV-treated eyes. Similarly, central retinal sections at PI-30 and PI-90 from AAVs and NPs exhibited GFP throughout the section (**Fig. 4b**, bottom and **Fig. 4c**), including in the periphery, not only at the site of injection (approximate location indicated by arrows in **Fig. 4b–c**). Consistent with our previous observations, no signal was detected with NP-CBA-GFP at PI-90. Significantly, GFP expression was still detected in fundus images at one year PI (**Fig. 4d**) in eyes injected with AAV2-CBA-GFP, AAV5-MOP-GFP, and NP-MOP-GFP indicating that both AAVs and NPs provide persistent gene expression with comparable ocular distribution patterns.

### Extraocular Expression of AAVs and NPs

**Figure 5:**
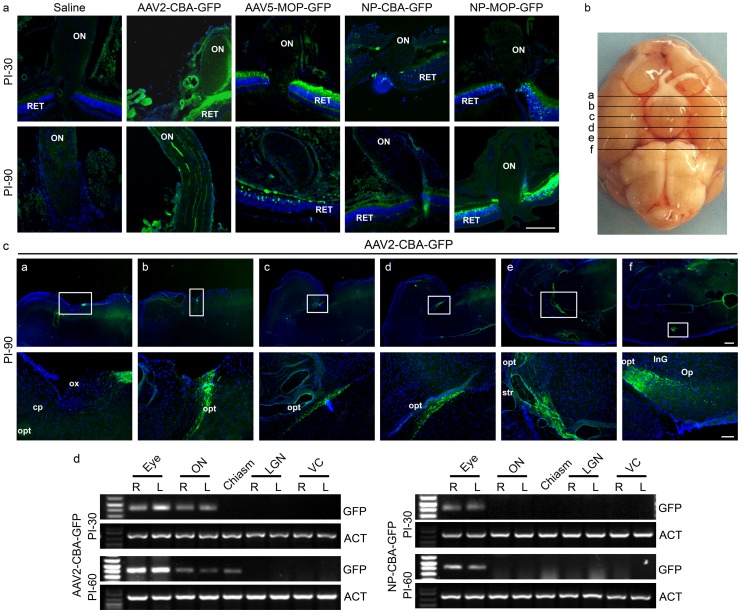
Subretinal injection of AAVs but not NPs leads to GFP expression in the brain. P30 Balb/C mice were subretinally injected bilaterally with NP-CBA-GFP, NP-MOP-GFP (6.9^11^ vg), AAV2-CBA-GFP, or AAV5-MOP-GFP (10^9^ vg). **a.** Native GFP fluorescence in the optic nerve (ON) was assessed by examination of central retinal cryosections. **b.** Whole brain schematic showing approximate regions where slices shown in (**c**), are captured. **c.** Transverse cryosections of whole brain were prepared for confocal microscopy at PI-90 days. Shown are representative low magnification (top row) and higher magnification (bottom row-magnification of box) images of native GFP fluorescence in the visual tract in animals injected with AAV2-CBA-GFP. **d.** GFP vector DNA was amplified from genomic DNA harvested from the eye, ON, optic chiasm, lateral geniculate nucleus (LGN), and visual cortex (VC). Shown are representative agarose gels from samples analyzed at PI-30 and PI-60 days. N = 8 (eye, ON, LGN, VC) and 4 (chiasm). cp: cerebral crus; InG: layers of superior colliculus; Op: optic nerve layer of the superior colliculus; opt: optic tract; ox: optic chasim; sox: supraoptic decussation; so: supraoptic. Scale bars **b.** 40 µm, **d.** 600 µm (top) and 160 µm (bottom). N values for **a, b** and **d** can be found in **Table 1**.

Delivery vehicles have differential ability to cross physiological barriers and transduce non-targeted cells, making careful assessment of any ectopic expression important. Although this can be partially controlled by employing tissue-specific promoters, inclusion of a ubiquitously expressed promoter makes assessment of extraocular expression paramount. The eye is separated from the systemic circulation by the blood-retinal barrier, but potential expression in the brain after ocular dosing is a distinct possibility, so we conducted two experiments to address this issue. We assessed 1) the presence of GFP protein by native fluorescence at PI-30, 60, 90, and 360, (**Fig. 5a,c** **, Fig. S3–S4** and **Table 1**), in the brain visual pathway in animals injected bilaterally with AAVs or NPs, and 2) the presence of vector DNA by PCR at PI-30 and 60 (**Fig. 5d** **,** **Table 1**) in the same series of tissues.

**Table 1:** GFP expression is restricted to the retina of NP-injected animals but is found in the brain of AAV-treated animals.

		AAV2-CBA-GFPCBA-GFP		AAV5-MOP-GFP		NP-CBA-GFP		NP-MOP-GFP	
***GFP Protein Expression (GFP Fluorescence)***									
**PI-30**	Cornea	−	(0/6)	−	(0/6)	−	(0/6)	−	(0/6)
	Lens	−	(0/6)	−	(0/6)	−	(0/6)	−	(0/6)
	Retina	+	(6/6)	+	(6/6)	+	(6/6)	+	(6/6)
	ON	+	(1/6)	−	(0/6)	−	(0/6)	−	(0/6)
	Chiasm	−	(0/3)	−	(0/3)	−	(0/3)	−	(0/3)
	LGN	−	(0/6)	−	(0/6)	−	(0/6)	−	(0/6)
	VC	−	(0/6)	−	(0/6)	−	(0/6)	−	(0/6)
**PI-60**	Cornea	−	(0/6)	−	(0/6))	−	(0/6)	−	(0/6)
	Lens	−	(0/6)	−	(0/6)	−	(0/6)	−	(0/6)
	Retina	+	(6/6)	+	(6/6)	+	(6/6)	+	(6/6)
	ON	+	(1/6)	−	(0/6)	−	(0/6)	−	(0/6)
	Chiasm	−	(0/3)	−	(0/3)	−	(0/3)	−	(0/3)
	LGN	−	(0/6)	−	(0/6)	−	(0/6)	−	(0/6)
	VC	−	(0/6)	−	(0/6)	−	(0/6)	−	(0/6)
**PI-90**	Cornea	−	(0/10)	−	(0/6)	−	(0/6)	−	(0/6)
	Lens	−	(0/10)	−	(0/6)	−	(0/6)	−	(0/6)
	Retina	+	(10/10)	+	(6/6)	−	(0/6)	+	(6/6)
	ON	+	(10/10)	−	(0/6)	−	(0/6)	−	(0/6)
	Chiasm	+	(1/5)	−	(0/3)	−	(0/3)	−	(0/3)
	LGN	+	(1/10)	−	(0/6)	−	(0/6)	−	(0/6)
	VC	+	(1/10)	−	(0/6)	−	(0/6)	−	(0/6)
**PI-360**	Cornea	−	(0/6)	−	(0/6)	−	(0/6)	−	(0/6)
	Lens	−	(0/6)	−	(0/6)	−	(0/6)	−	(0/6)
	Retina	+	(6/6)	+	(6/6)	−	(0/6)	+	(6/6)
	ON	+	(6/6)	−	(0/6)	−	(0/6)	−	(0/6)
	Chiasm	+	(1/3)	−	(0/3)	−	(0/3)	−	(0/3)
	LGN	+	(2/6)	−	(0/6)	−	(0/6)	−	(0/6)
	VC	+	(2/6)	−	(0/6)	−	(0/6)	−	(0/6)
***GFP Vector DNA***									
**PI-30**	Eye	+	(8/8)			+	(8/8)		
	ON	+	(6/8)			−	(0/8)		
	Chiasm	−	(0/4)				(0/4)		
	LGN	−	(0/6)			−	(0/6)		
	VC	−	(0/6)			−	(0/6)		
**PI-60**	Eye	+	(8/8)			+	(8/8)		
	ON	+	(6/8)			−	(0/8)		
	Chiasm	+	(3/4)			−	(0/4)		
	LGN	−	(0/8)			−	(0/8)		
	VC	−	(0/8)			−	(0/8)		

GFP expression by native fluorescence and vector DNA detection by qPCR after bilateral subretinal injection of AAVs and NPs. Values expressed as (# expressing/# examined).

Consistent with promoter specificity, GFP fluorescence was not seen in the optic nerve (ON) (**Fig. 5a**) or brain (**Table 1**) in any animals treated with MOP-containing vectors (AAV or NP), although retinal expression was seen with these vectors up to PI-360 (**Table 1**). GFP was observed in the ON of 1/6 AAV2-CBA-GFP treated animals at PI-30 and PI-60 (**Fig. 5a** **,** **Table 1**). In contrast, no GFP was detected in the ON of NP-CBA-GFP treated animals at PI-30 or PI-60 (**Fig. 5a** top, **Table 1**), although retinal expression was seen in NP-CBA-GFP treated animals at these timepoints. At PI-90, ON expression was observed in all 10 AAV2-CBA-GFP treated eyes while no GFP was detected in the ON of NP-CBA-GFP counterparts. At PI-30 and PI-60, vector DNA was amplified in the eyes of all AAV2-CBA-GFP and NP-CBA-GFP treated eyes, and in the ON of 6/8 AAV2-CBA-GFP eyes, but none of the ONs of NP-CBA-GFP eyes (**Fig. 5d** **,** **Table 1**).

At PI-30 and PI-60, no GFP fluorescence was detected in the optic chiasm, lateral geniculate nucleus (LGN) or visual cortex (VC) in eyes injected with any of the four treatments (**Table 1**). However, in 3/4 AAV2-CBA-GFP animals examined, vector DNA was amplified from the optic chiasm at PI-60 (**Fig. 5d** **,** **Table 1**), portending future gene expression there. Indeed at PI-90, animals injected with AAV2-CBA-GFP exhibited GFP expression in the optic chiasm (1/5), LGN (1/10) and VC (1/10) (**Fig. 5b–c** **,** **Table 1**). Similarly, at PI-360; one-third of AAV2-CBA-GFP treated animals exhibited GFP expression in the optic chiasm, LGN, and VC (**Table 1** **, Fig. S3**). Neither vector DNA nor GFP expression was detected in the ON/brain in any NP-treated animals (**Table 1** **, Fig. S4**). In AAV2-treated animals extraocular expression was restricted to the visual pathway and no GFP was found in other regions of the brain. Furthermore, we did not observe GFP expression (by qRT-PCR) in non-CNS organs including the heart, liver and lungs at PI-360 (data not shown) from any AAV2-CBA-GFP or NP-CBA-GFP-treated animals.

## Discussion

Here we conduct the first side-by-side comparison of CK30PEG NPs and AAVs carrying matched expression cassettes. In our dose-response assay we determined that NPs are less efficient per vg than AAVs, but that NPs produce gene expression levels similar in scale to those generated by AAV. Although self-complementary AAV vectors bypass the requirement for viral second-strand DNA synthesis, and therefore have a faster onset of gene expression than conventional AAVs, NPs still had earlier onset of expression (PI-2 vs. PI-7). Expression levels were similar for AAVs and NPs at PI-14, but at subsequent timepoints, GFP levels from AAVs were higher than those from NPs. However, if dose equivalency had been chosen based on dose-response results from PI-30, then no difference in PI-120 expression levels between these delivery systems might have been observed. Overall, our data from the dose-response studies indicate that NP-driven gene expression can be modulated by altering the dose and suggest that higher levels of NP-based expression can be achieved if needed. Importantly, the NP doses that we use, although higher than the doses of AAV, are stable, and easy to manufacture [15], [21], [24]. Although future studies may examine the immune response to nanoparticles in more depth, importantly, we have demonstrated that the doses we use here are well tolerated and do not induce an inflammatory response after delivery to the eye [15], [21], [24]. In addition, we have shown that they are well tolerated even after repeat injection [24], a key feature if multiple dosing is therapeutically required.

Interestingly, although AAV2-CBA-GFP was expressed in all retinal layers while AAV5-MOP-GFP expression was limited to photoreceptors, these two treatments generated similar levels of GFP message and protein. This may be because AAV5 was reported to drive photoreceptor gene expression at higher levels than AAV2 [28], thus leading to similar expression levels in animals treated with photoreceptor specific (AAV5-MOP) and ubiquitously expressed (AAV2-CBA) AAVs. However, we also observed that at PI-14 and PI-30 NP-CBA-GFP and NP-MOP-GFP exhibited similar levels of GFP expression suggesting that promoter strength also contributes to expression levels independent of delivery strategy.

Although persistent gene expression is critical for successful ocular gene therapy and has been elusive for non-viral vectors, here we show that NPs do not suffer from transient expression. Neither NP-MOP-GFP, AAV2-CBA-GFP, nor AAV5-MOP-GFP exhibit significant decreases in gene expression between PI-30 and PI-120 and fundus images and retinal sections examined at PI-360 demonstrate long-term expression in NP-MOP-GFP and AAV treated animals. However, it is critical to observe that the vector content/delivery vehicle affected the persistence of gene expression. AAV2-CBA-GFP injected animals express GFP for up to one year, while NP-CBA-GFP was silenced by PI-90. It is not clear why the CBA promoter drives persistent gene expression when delivered in an AAV, but transient expression when delivered as a NP, but several possibilities exist. Firstly, the DNA content of the two delivery systems is different. Although both the NPs and the AAV were generated from matched ITR plasmids, the NPs contain the entire plasmid while AAV production results in virions carrying only the expression cassette. Prokaryotic plasmid backbone element can influence gene expression [19], [36], and may promote NP-CBA-GFP silencing. A second contributing factor may be differential methylation or epigenetic state between AAV and NP DNA arising from the biological source of the DNA: bacteria for NPs and HEK cells for AAV. Finally, NPs remain episomal, but AAVs can be episomal or integrated [10], [37]. Some of the AAVs may have integrated into the genome thereby promoting different regulation of gene expression than the NPs. Despite these differences between the delivery systems, our observation that NP-MOP-GFP can generate persistent retinal gene expression indicates that the delivery method per se is not responsible for the lack of long-term gene expression from NP-CBA-GFP.

These data emphasize the importance of proper selection and testing of vector elements, and it has been well established that the same gene with different promoters may have different therapeutic effects and safety profiles [19], [38], [39], [40], [41]. For example, we previously demonstrated that NPs carrying pZeo-CMV-GFP had high expression in the eye and lung at PI-2 but were silenced thereafter [38], [42]. In contrast, in keeping with what we show here with NP-MOP-GFP, we have also observed persistent gene expression from other vector/promoter combinations. NPs carrying pcDNA-MOP-NMP (NMP-normal mouse peripherin/retinal degeneration slow) drove photoreceptor gene expression for up to 10 months (longest timepoint examined) [15], and our ongoing work has shown photoreceptor and RPE expression for up to 15 months and 2 years, respectively (unpublished data) with a variety of vectors. In the past, tissue-specific promoters have been successfully used not only in NPs but also in AAVs to target multiple retinal cell types [29], [30], [43]_ENREF_51. The need for this tissue specificity in gene therapy vectors is highlighted by our observation that subretinally injected AAV leads to GFP expression in the visual pathways of the brain only when a ubiquitous promoter is used. It is difficult to conclusively say whether NPs could travel to the brain and drive gene expression since brain expression from AAV2-CBA-GFP was not observed until PI-90 days, a timepoint at which NP-CBA-GFP was silenced. However, our results from DNA amplifications (**Table 1**) suggest that subretinally injected NPs do not leave the eye. In contrast to AAV2-CBA-GFP DNA which was detected in the eye, ON, and optic chiasm at PI-60 days, we detected NP-CBA-GFP DNA only in the eye.

The issue of AAV2-based brain expression after subretinal injection has been extensively studied yet remains controversial. Although subretinal delivery of rAAV2-RPE65 did not lead to vector or gene expression in the visual pathways in the brain of RPE65-mutant dogs, that study was only maintained for three months [2], a timepoint at which we just began to observe GFP expression in the mouse brain. In contrast, other groups showed that intravitreal or subretinal injection of AAV drives expression in the brain along the visual pathway in rat, dog, mouse, and pig [44], [45], [46], [47]_ENREF_53_ENREF_55. Delivery of AAV to the brain by ocular injection has even been used to mediate improvements in mouse models of lysosomal storage disease [48]. Here we observe gene expression consistent with anterograde axonal transport from retinal ganglion cells (i.e. expression in ON, optic chiasm, and LGN) as well as expression consistent with trans-synpatic transport of the virus (expression in the VC). While others have also observed trans-synaptic transport of recombinant, replication deficient AAVs [46], [48], the mechanisms that underlie this process are not understood. Ectopic expression and transmission of the virus from the target cell to other cells may be harmful [49] and are undesirable from a regulatory standpoint.

In this side-by-side comparison study of AAVs and NPs we demonstrate that CK30PEG NPs can safely drive persistent gene expression (up to 1 year) after subretinal injection in adult mice. In addition, in contrast to AAVs, which were detected in the visual pathways of the brain, NPs remained in the eye. These NPs have several benefits for intraocular use; not only are they safe and non-toxic to the eye [15], [21], [24], but they have a much larger vector capacity than AAVs [26], features that make them a highly clinically relevant complement to AAV for ocular gene therapy and an excellent option for the delivery of genes that are too large for AAV.

## Materials and Methods

### Ethics Statement and Animal Studies

All experiments and animal maintenance were approved by the University of Oklahoma Health Science Center Institutional Animal Care and Use Committee (IACUC) and adhered to the ARVO Statement for the Use of Animals in Ophthalmic and Vision Research. Balb/c mice were obtained from Harland Laboratories and used for all experiments.

### Plasmids and Vectors

AAV vector backbone plasmid pscCBA-GFP was kindly provided by Dr. Arun Srivastava, Department of Medicine at the University of Florida; pscMOP500-GFP was kindly provided by Dr. William Hauswirth, Department of Ophthalmology at the University of Florida, Gainesville, FL. Both DNA vectors were sent to Aldevron, Inc. (Fargo, ND) to generate clinical grade, endotoxin-free DNAs which were then used for NP compaction. The MOP500 (−385 to +86) promoter was chosen based on our previous experience and supporting data from other investigators [29]. The CBA promoter is 544 bp and has been used extensively for gene therapy [10], [16], [31].

### Recombinant AAV Vector Production

Viral particles used in the present study were scAAV2-CBA-GFP (hereafter AAV2-CBA-GFP, 5770 bp) and scAAV5-MOP500-GFP (hereafter AAV5-MOP-GFP, 5781 bp) and were generated as previously described using the same ITR plasmids mentioned above [10], [30], [31]. Briefly, 293T cells at ∼70% confluency were co-transfected with pAdeno-helper plasmid and AAV-Rep-Cap-helper plasmids pRC2 and pRC5, respectively, which supply all necessary helper functions as well as *rep* and *cap* gene products in *trans*. Vectors were purified by benzonase treatment of cell lysates, iodixanol step gradient centrifugation, and HiTrap Q HP (for AAV5) or HiTrap SP HP (for AAV2) columns (GE Healthcare Life Sciences, Piscataway, NJ). Physical titers (vector genome numbers, vg) of purified vectors were determined by DNA slot-blot analyses.

### DNA-nanoparticle Preparation

DNA NPs were formulated by mixing plasmid DNA with CK_30_PEG10K, a 30-mer lysine peptide with an N-terminal cysteine that is conjugated via a maleimide linkage to 10 kDa polyethylene glycol, as previously described [26]. The polycation contained an acetate counterion at the time of DNA mixing which produces rod-shaped NPs. NPs were concentrated to 4.3 mg/ml of DNA in saline and processed by tangential flow filtration to remove excess CK_30_PEG10k. DNA NPs were characterized by a panel of quality control tests, including transmission electron microscopy (NP size and shape), turbidity and saline sedimentation analyses (colloidal stability), serum stability test (protection of DNA from nucleases), endotoxin measurements, and gel analysis (DNA integrity). DNA NPs containing either CBA-GFP (NP-CBA-GFP) or MOP-GFP (NP-MOP-GFP) met all quality control assay standards. NP concentration was determined by measuring DNA concentration and converting to copy number based on the size of the vector.

### Subretinal Injections

Subretinal injection was performed as previously described [15]. Briefly, wide type balb/c mice at postnatal 30 (P30) were anesthetized by an intramuscular injection of 80 mg/kg ketamine and 14 mg/kg xylazine (Butler Schein Animal Health, Dublin, OH). After eye dilation with cyclopentolate, a sterile 28-gauge needle (BD Biosciences, Franklin Lakes, NJ) was used to puncture the cornea, avoiding any contact with the lens. A 33-gauge blunt-end needle attached to a 10 µl Nanofil® syringe (World Precision Instruments, Sarasota FL) was then inserted into the puncture under an operating microscope (Carl Zeiss Surgical, Inc., NY). One µl of solution containing either uncompacted (naked) vectors, NPs, AAV, or saline (vehicle) was delivered into the subretinal space in the temporal central region. In one set of experiments, the injection volume was 1.45 µl. Gonak Hypromellose eye drops (2.5%; Akorn, Inc., Buffalo Grove, IL) were applied to the eyes of injected animals immediately after the procedure. Animals were closely monitored after the procedure and those with complications, such as subretinal bleeding, damage of the lens, intraocular infection, or subsequent development of cataracts were excluded from analysis (less than 15%).

### Quantitative qRT-PCR Analysis

qRT-PCR was performed as previously described [15], [16], [38]. Briefly, total RNA was extracted from the tissue of a single eye using Trizol reagent (Invitrogen Inc., Carlsbad, CA) and then 2 µg of isolated RNA was treated with RNAse-free-DNAse I (Promega Inc., San Luis Obispo, CA). Reverse transcription (RT) was performed using an oligo-dT primer and Superscript III reverse transcriptase (Invitrogen Inc., Carlsbad, CA). A no-RT (contains everything except the reverse transcriptase) sample was used as a control for any residual compacted DNA or genomic DNA contaminants. qRT-PCR was performed in triplicate on each cDNA sample using a Bio-Rad C1000 Thermal Cycler (SYBR Green) and ΔcT values were calculated against the mouse β-actin housekeeping gene. Three independent qRT-PCR experiments for each set of samples were performed and values were averaged. Relative gene expression values were determined using the following formula: Relative Expression = 2^−ΔcT^, where ΔcT = (gene CT− β-actin CT). At least six injected (including saline injected controls) and six uninjected eyes from each treatment group at each of the scheduled time-points were analyzed. We did not see any signals in the uninjected or saline injected samples nor in the no-RT controls. Agarose gel electrophoresis and disssociation curve analysis were also performed on all PCR products to confirm proper amplification. The primers used in this study were as follows: GFP, forward: 5′- TACATCATGGCCGACAAGCA-3′; reverse: 5′- AACTCCAGCAGGACCATGTG-3′; mouse actin, forward: 5′- TGTTACCAACTGGGACGACA-3′; reverse: 5′- CTTTTCACGGTTGGCCTTAG-3.

### Immunoblotting

Retinas were homogenized and lysed in lysis buffer (50 mM Tris pH 7.8, 100 mM NaCl, 5 mM EDTA, 0.05% SDS, 1% TX-100, 2.5% glycerol, and 1 mM PMSF). Protein concentrations were determined using the Bio-Rad protein assay kit (Bradford assay, Bio-Rad, Hercules, CA). Equal amounts of total protein were separated using 10% SDS-PAGE, and electrotransferred to PVDF membrane (Millipore, Inc, Billerica, MA). Membranes were blocked with 5% non-fat milk in TBST, then incubated in anti-GFP (1∶1000, A11122, rabbit monoclonal, Invitrogen, Inc.) for 1 hr at room temperature. Membranes were then incubated with HRP conjugated anti-rabbit IgG antibody at 1∶25,000 for 1 hr at room temperature, and visualized using SuperSignal West Dura Extended Duration Substrate (Thermo Scientific, Rockford, IL) according to the manufacturer’s guidelines. Densitometric analysis was conducted using Kodak Image Station 4000R Software (Carestream Health, Inc., Rochester, NY) and the pixel densities in each band were normalized to the amount of β-actin in each lane. Since WT mice do not express GFP, to control for variations in GFP and β-actin blot exposure times from experiment to experiment, after normalizing to β-actin, GFP levels were expressed as a ratio to the amount of GFP found in GPI-GFP transgenic mice [50], a positive control which was included in every blot.

### Immunohistochemistry

Tissue fixation and sectioning were performed as previously described [16], [38]. Briefly, eyes from mice at different time points post-injection were enucleated and fixed with phosphate-buffered saline containing 4% paraformaldehyde at 4°C for 1 hr. The cornea and lens were removed and the eye was returned to fixative for an additional two hours. Eyecups were then sequentially immersed in 10%, 20%, and 30% (w/v) sucrose solutions. Each eyecup was embedded in M1 embedding medium (Thermo Electron Corporation, PA) and frozen on dry ice; frozen sections (10 µm thickness) aligned with the vertical meridian were cut with a cryostat (Leica, Buffalo Grove, IL) and collected on precleaned Superfrost-plus® microscope slides (Fisher Scientific). The entire eye was sectioned, and every tenth section was collected. For study of GFP distribution, central retinal cross sections (i.e. containing the optic nerve head) were examined. For immunohistochemistry (IHC), sections were blocked in 5% BSA, 3% Triton X-100 in PBS, then mouse monoclonal anti-rod opsin 1D4 (kindly provided by Dr. Robert. S. Molday from University of British of Columbia, Vancouver, Canada), goat anti-s opsin (Santa Cruz Biotechnology, Inc.), rat monoclonal anti-F4/80 to macrophages (Abcam, USA; 1∶500) in 5% BSA, 3% Triton X-100 in PBS were used as described in the figures. Appropriate Alexa Flour 555 or 647 secondary antibodies (Invitrogen, Inc.) were used, in 5% BSA, 3% Triton X-100 in PBS. Slides were mounted using Vectashield with DAPI, (Vector Laboratories, Burlingame, CA). Imaging was performed using a spinning disk confocal microscope (BX62 Olympus, Japan). To control for normal retinal autofluorescence, images were captured at equivalent exposure times from control eyes.

### Color Fundus Photography

Micron III imaging system (Phoenix, Research Laboratories, Inc., Pleasanton, CA) was used to capture brightfield and green fluorescent fundus images. Mice were anesthetized and eyes dilated as described above. The cornea was covered with one drop of 2.5% Gonak Hypromellose to reduce corneal scattering, and the objective was positioned on the surface of the eye. Focus and illumination were adjusted during examination and images were captured using the Streampix Software (Phoenix Research Laboratories).

### Statistics

To determine whether statistically significant differences in mean mRNA and protein levels existed between AAV and NP-treated eyes in timecourse experiments, results were tested using two-way ANOVA (time and treatment as variables) with Bonferroni’s post-hoc tests. For dose-response studies, mean levels in AAV-treated eyes were compared to those in NP-treated eyes using two-tailed, unpaired Student’s t-test.

## Supporting Information

Figure S1 **Subretinal injection of AAVs and NPs did not induce macrophage infiltration.** P30 Balb/C mice were subretinally injected with NP-CBA-GFP, NP-MOP-GFP (6.9^11^ vg), AAV2-CBA-GFP, or AAV5-MOP-GFP (10^9^ vg). Top: Cryosections collected at PI-14 were labeled with antibodies against the macrophage marker F4/80 (red). Bottom: Eyes injected with *B. Cereus* were used as positive controls for inflammation and toxic intraocular responses. Uninjected mice were used as negative controls. Scale bar: 20 µm.
(TIF)

Figure S2 **GFP is expressed for up to one year in AAV and NP treated animals.** Balb/C mice were subretinally injected at P30 with Naked-CBA-GFP, Naked-MOP-GFP, NP-CBA-GFP, NP-MOP-GFP (all at 4.3 µg/µl or 6.9^11^ vg), AAV2-CBA-GFP, or AAV5-MOP-GFP (at 10^9^ vg). GFP distribution was examined *in vivo* by brightfield/GFP fundus imaging at the indicated ages. Shown are fundus images with the green channel only taken from **Fig. 5** to facilitate interpretation.
(TIF)

Figure S3 **Distribution of GFP expression in the brain at 1 year PI.** Transverse cryosections of whole brain were prepared for confocal microscopy at PI-360 days. To accommodate the size of the brain section, images in **a-f** and **b’-f’** are composites of two adjacent frames. The entire section fit in one image frame in **a’**. Strong GFP expression was detected in animals treated with AAV2-CBA-GFP and expression was restricted to the vision pathway. Left panels, brightfield images, right panels, native GFP fluorescence. Lowercase letters correspond approximately with the brain schematic shown in **Fig. 5b**. cp: cerebral crus; InG: layers of superior colliculus; Op: optic nerve layer of the superior colliculus; opt: optic tract; ox: optic chasim; sox: supraoptic decussation; so: supraoptic. Scale bars, 400 µm.
(TIF)

Figure S4 **No expression of NP-CBA-GFP is found in the brain.** Transverse cryosections of whole brain were prepared for confocal microscopy at PI-90 days. Shown are representative low magnification (left column) and higher magnification (right column) images of native GFP fluorescence in the visual tract (as presented in **Fig. 5b**) in animals injected with NP-CBA-GFP. Scale bars 600 µm (left) and 160 µm (right). No GFP fluorescence was detected in the brain of any NP injected animals. N values can be found in **Table 1**.
(TIF)
