# Supplementary figures and images for: Comparative Analysis of DNA Nanoparticles and AAVs for Ocular Gene Delivery
Source: PLoS One. 2012 Dec 18;7(12):e52189. doi: 10.1371/journal.pone.0052189 (PMC3525534; doi:10.1371/journal.pone.0052189)

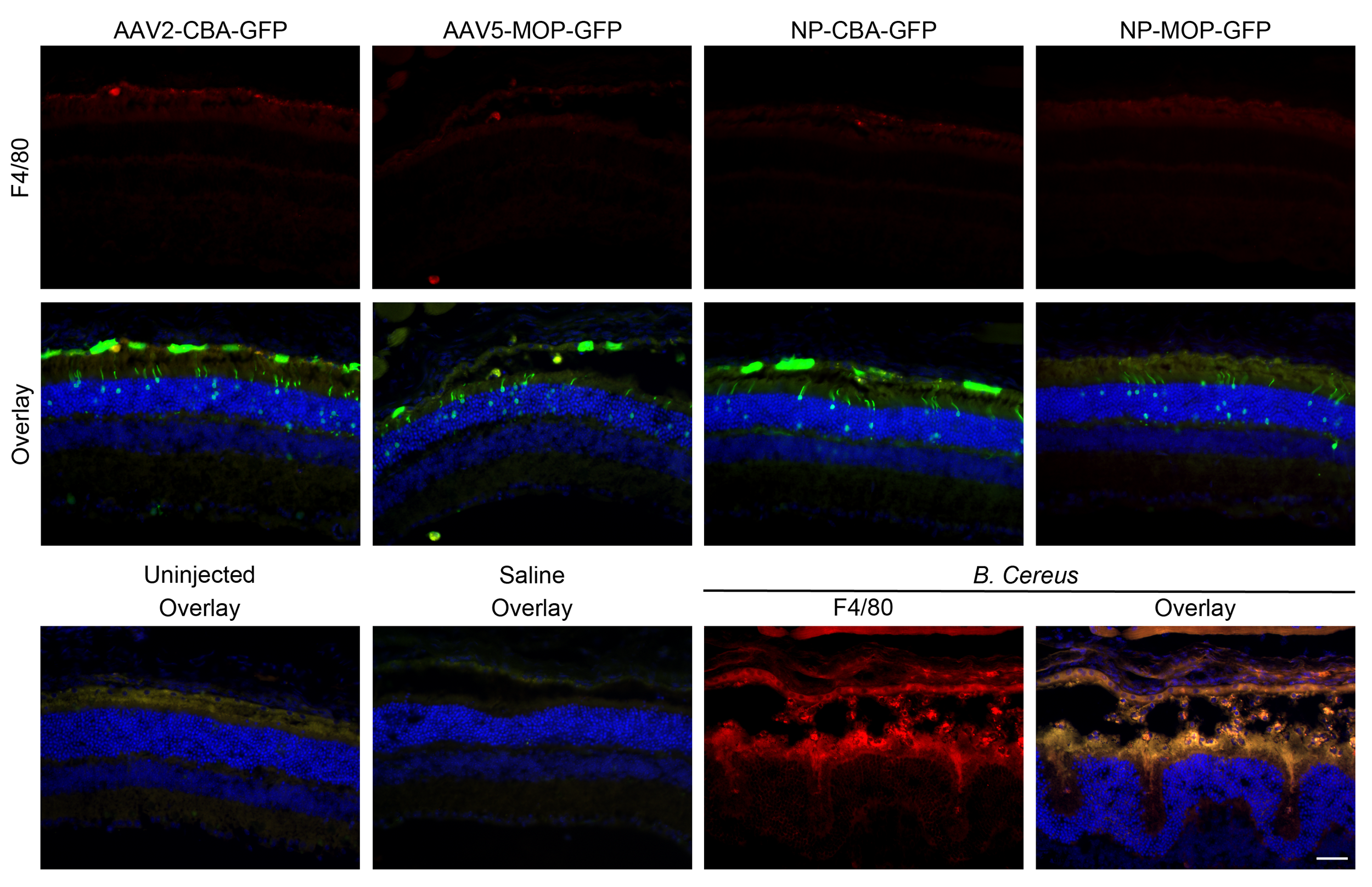

Supplement: Figure S1 — Subretinal injection of AAVs and NPs did not induce macrophage infiltration. P30 Balb/C mice were subretinally injected with NP-CBA-GFP, NP-MOP-GFP (6.911 vg), AAV2-CBA-GFP, or AAV5-MOP-GFP (109 vg). Top: Cryosections collected at PI-14 were labeled with antibodies against the macrophage marker F4/80 (red). Bottom: Eyes injected with B. Cereus were used as positive controls for inflammation and toxic intraocular responses. Uninjected mice were used as negative controls. Scale bar: 20 µm. (TIF) [file pone.0052189.s001.tif]

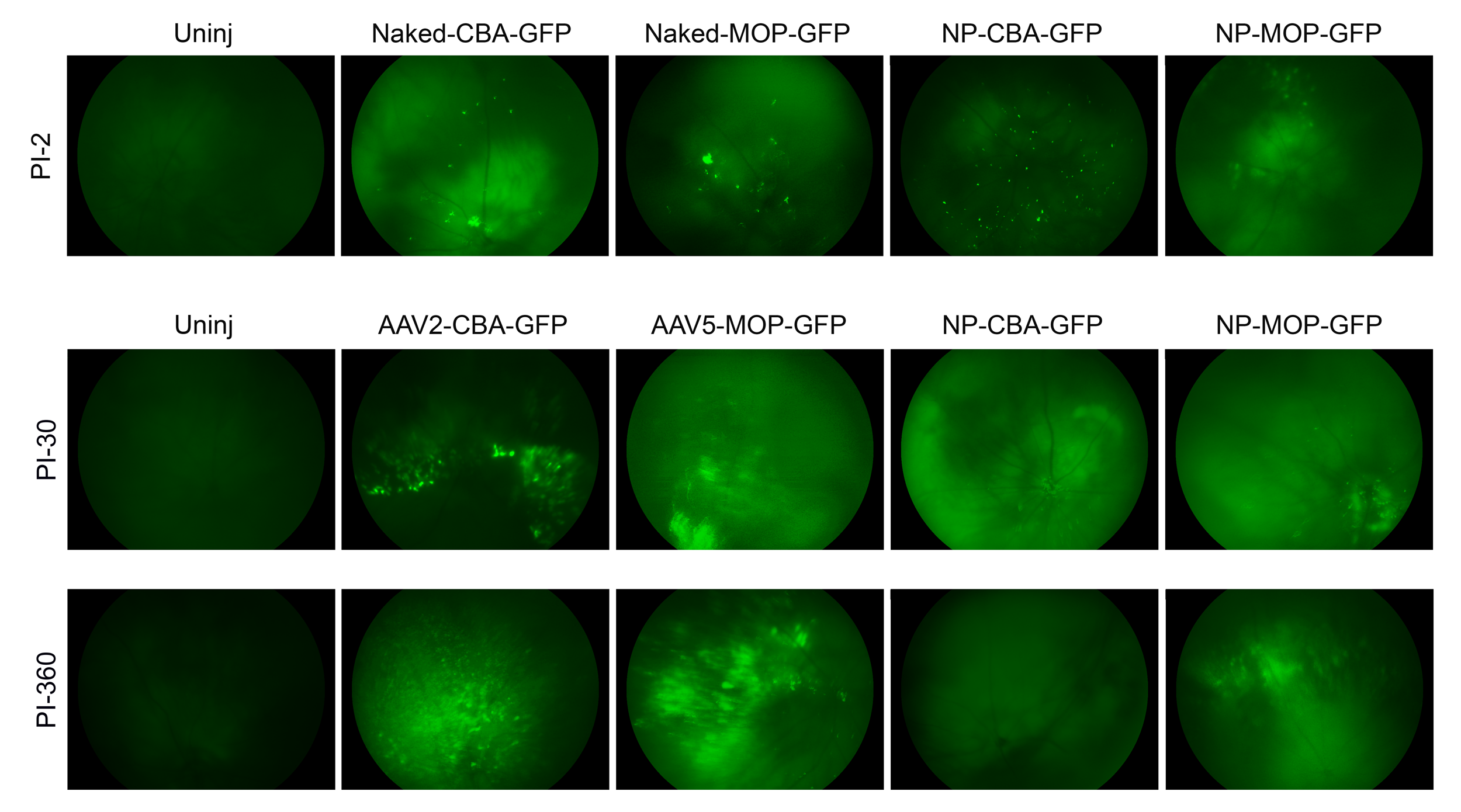

Supplement: Figure S2 — GFP is expressed for up to one year in AAV and NP treated animals. Balb/C mice were subretinally injected at P30 with Naked-CBA-GFP, Naked-MOP-GFP, NP-CBA-GFP, NP-MOP-GFP (all at 4.3 µg/µl or 6.911 vg), AAV2-CBA-GFP, or AAV5-MOP-GFP (at 109 vg). GFP distribution was examined in vivo by brightfield/GFP fundus imaging at the indicated ages. Shown are fundus images with the green channel only taken from Fig. 5 to facilitate interpretation. (TIF) [file pone.0052189.s002.tif]

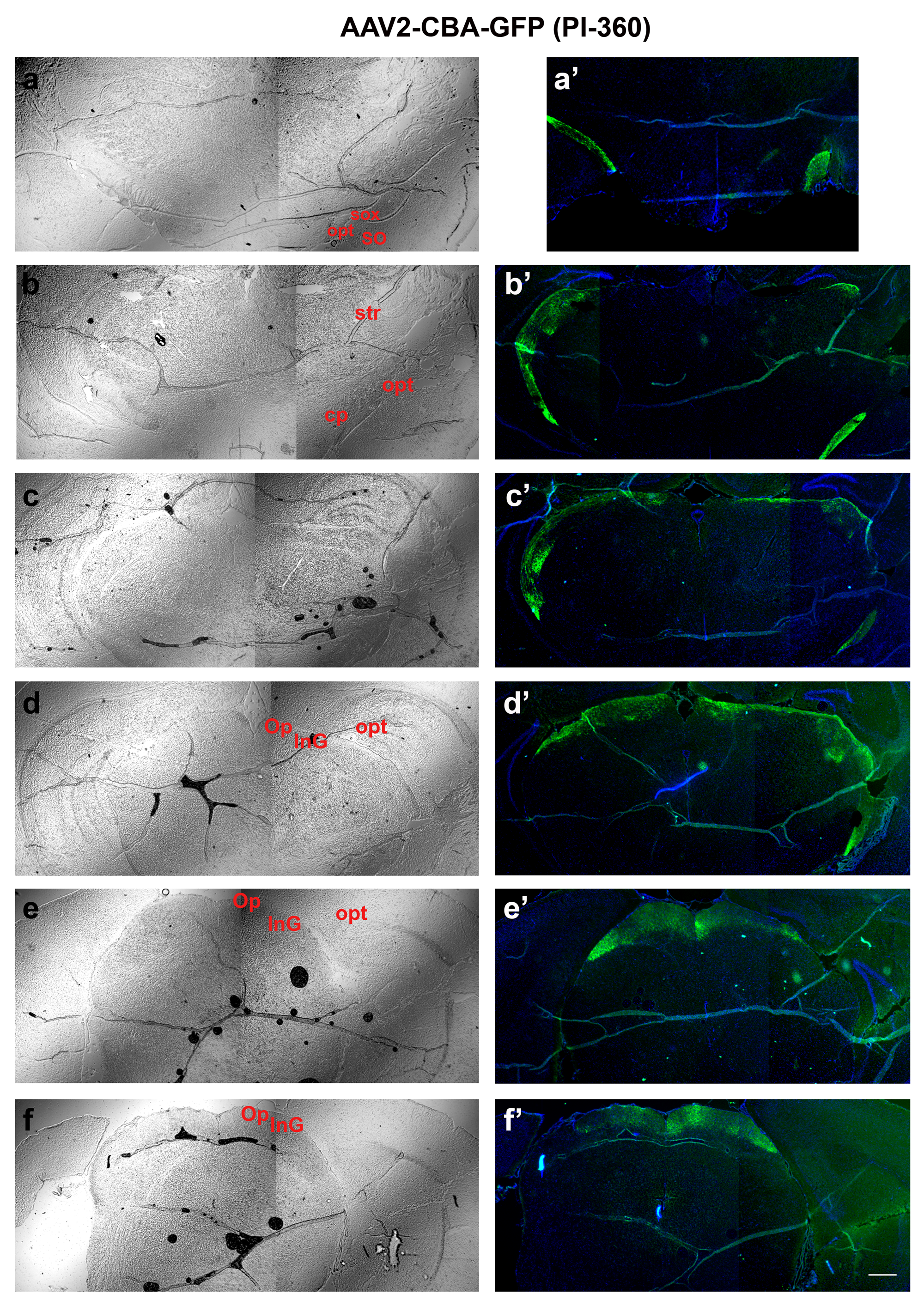

Supplement: Figure S3 — Distribution of GFP expression in the brain at 1 year PI. Transverse cryosections of whole brain were prepared for confocal microscopy at PI-360 days. To accommodate the size of the brain section, images in a-f and b’-f’ are composites of two adjacent frames. The entire section fit in one image frame in a’. Strong GFP expression was detected in animals treated with AAV2-CBA-GFP and expression was restricted to the vision pathway. Left panels, brightfield images, right panels, native GFP fluorescence. Lowercase letters correspond approximately with the brain schematic shown in Fig. 5b . cp: cerebral crus; InG: layers of superior colliculus; Op: optic nerve layer of the superior colliculus; opt: optic tract; ox: optic chasim; sox: supraoptic decussation; so: supraoptic. Scale bars, 400 µm. (TIF) [file pone.0052189.s003.tif]

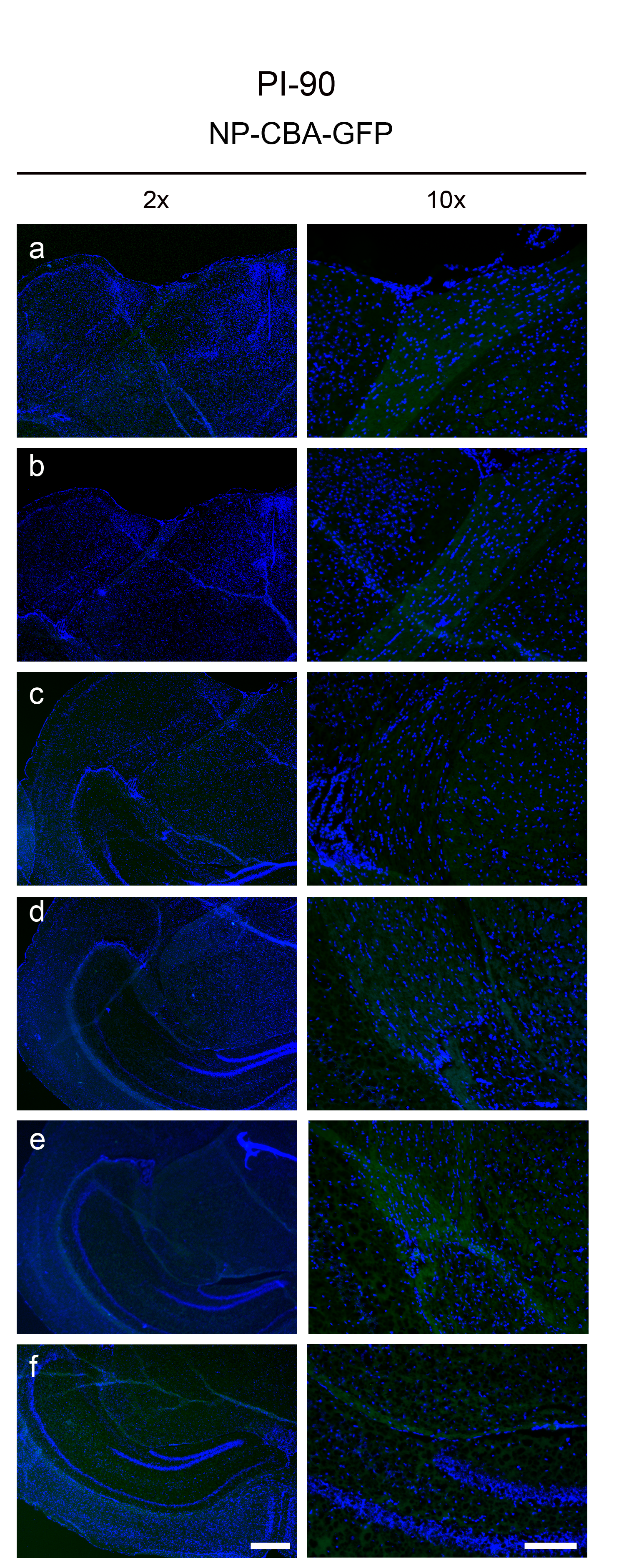

Supplement: Figure S4 — No expression of NP-CBA-GFP is found in the brain. Transverse cryosections of whole brain were prepared for confocal microscopy at PI-90 days. Shown are representative low magnification (left column) and higher magnification (right column) images of native GFP fluorescence in the visual tract (as presented in Fig. 5b ) in animals injected with NP-CBA-GFP. Scale bars 600 µm (left) and 160 µm (right). No GFP fluorescence was detected in the brain of any NP injected animals. N values can be found in Table 1 . (TIF) [file pone.0052189.s004.tif]
